# Dual-Mode Data Collection for Periodic and Urgent Data Transmission in Energy Harvesting Wireless Sensor Networks

**DOI:** 10.3390/s25082559

**Published:** 2025-04-18

**Authors:** Ikjune Yoon

**Affiliations:** Division of AI Computer Science and Engineering, Kyonggi University, Suwon-si 16227, Republic of Korea; ijyoon@kyonggi.ac.kr

**Keywords:** wireless sensor networks, energy harvesting, dual-mode, periodic data, urgent data

## Abstract

Wireless Sensor Networks (WSNs) are widely used for environmental data collection; however, their reliance on battery power significantly limits network longevity. While energy harvesting technologies provide a sustainable power solution, conventional approaches often fail to efficiently utilize surplus energy, leading to performance constraints. This paper proposes an energy-efficient dual-mode data collection scheme that integrates Long Range Wide Area Network (LoRaWAN) and Bluetooth Low Energy (BLE) in an energy-harvesting WSN environment. The proposed method dynamically adjusts sensing intervals based on harvested energy predictions and reserves energy for urgent data transmissions. Urgent messages are transmitted via BLE using multi-hop routing with redundant paths to ensure reliability, while periodic environmental data is transmitted over LoRaWAN in a single hop to optimize energy efficiency. Simulation results demonstrate that the proposed scheme significantly enhances data collection efficiency and improves urgent message delivery reliability compared to existing approaches. Future work will focus on optimizing energy consumption for redundant urgent transmissions and integrating error correction mechanisms to further enhance transmission reliability.

## 1. Introduction

Wireless Sensor Networks (WSNs) are widely used to collect large amounts of environmental data [1,2,3]. Although traditional WSNs operate wirelessly, facilitating easy installation and operation, they suffer from significant limitations due to battery capacity constraints, leading to a limited lifetime and maintenance challenges. Previous methods aimed at increasing node lifetime by reducing energy consumption [4,5] still cannot fundamentally resolve this limitation. Therefore, recent research has explored energy harvesting and wireless power transfer (WPT) techniques to permanently extend the lifespan of sensor nodes [6,7].

Energy harvesting allows sensor nodes to collect energy from the environment, theoretically offering infinite operational lifetime [8,9,10]. While most existing systems store harvested energy in rechargeable batteries, these are limited by their finite number of charge/discharge cycles, raising concerns about long-term maintenance. To address this limitation, supercapacitors have been proposed as alternative energy storage devices due to their extremely high durability, virtually unlimited charge/discharge cycles, and fast charging capability. However, supercapacitors generally suffer from low energy density and significantly lower storage capacity compared to batteries. As a result, they may not be suitable for applications that require accumulating harvested energy over time and using it steadily regardless of sunlight availability or time, such as solar-powered systems operating through the night or during cloudy conditions. Regardless of the storage medium, surplus harvested energy that exceeds the capacity of the energy storage device often remains unused, motivating further research on enhancing system performance by efficiently utilizing this surplus energy. In particular, techniques that adjust transmission routes [11,12,13] or control the amount of sensored data [14,15,16] based on energy status have been extensively studied.

Traditional WSNs typically use low power wireless personal area network (LPWPAN) communication technologies such as ZigBee or Bluetooth Low Energy (BLE) to transmit sensing data to a sink node using multi-hop methods. These approaches can cause rapid energy depletion in relay nodes as the network size expands, leading to reduced network lifespan. To overcome this limitation, studies have been conducted utilizing low-power wide area network (LPWAN) communication technologies such as Long Range (LoRa) or SigFox [17,18]. These LPWAN technologies have very low data rates and high transmission latency; however, they can achieve transmission distances of several kilometers, allowing sensor nodes to transmit data to a sink node in a single hop. This approach is suitable for intermittently transmitting small amounts of sensing data; however, it is not appropriate for urgent transmission of large amounts of data due to its long Time on Air (ToA). Therefore, recent studies have focused on techniques that utilize both LPWPAN and LPWAN technologies together in a single sensor device [19,20,21,22,23,24].

If conventional sensing period adjustment methods [16,25] are applied to efficiently utilize energy, all available energy might be consumed for periodic sensing data collection, resulting in energy depletion and the inability to transmit data during urgent situations. In particular, this phenomenon becomes more severe when transmitting large-sized data such as ambient sound, images, and videos from the site. Thus, an energy utilization strategy suitable for these applications is required.

In this paper, we propose an efficient energy management method to evenly increase the amount of data gathered by nodes in WSNs where a sensor node equipped with two transmission modules, LoRa and BLE, periodically collects environmental data and intermittently transmits a large amount of urgent data to a sink node. The sensor nodes employed in this study utilize batteries, which are recharged through energy harvested from the surrounding environment. Although supercapacitors are often used in maintenance-free systems due to their long cycle life, their relatively low energy storage capacity makes them less suitable for our proposed approach, which requires accumulating harvested energy over time to support both periodic and urgent transmissions. For this reason, we adopted batteries as the energy storage medium to ensure adequate energy availability and operational flexibility. Periodically collected environmental data are transmitted directly to the sink node in a single hop using Long Range Wide Area Network (LoRaWAN), a low-power wireless wide-area network technology, to enhance energy efficiency. To efficiently manage energy, sensor nodes reserve energy for the transmission of urgent messages that may occur unpredictably. Nodes allocate available energy within a range that prevents both energy depletion and overcharging, adjusting their data transmission intervals accordingly. When urgent messages occur, nodes transmit large amounts of data rapidly using BLE through a multi-hop route to the sink node. Additionally, to mitigate scenarios where intermediate relay nodes may fail or data transmission errors occur, duplicated data are transmitted simultaneously over multiple redundant paths. In such cases, nodes utilize reserved energy for urgent data transmission and subsequently adjust their sensing intervals to replenish the consumed energy. This strategy enables sensor nodes to reliably transmit urgent data while maximizing the collection of periodic sensing data.

The remainder of the paper is structured as follows: Section 2 presents related work; Section 3 details the proposed scheme; Section 4 evaluates performance through simulations; and Section 5 concludes the paper and outlines future research directions.

## 2. Related Work

Various approaches combining different communication technologies have been studied to enhance efficiency in WSNs. Truong et al. [19] analyzed the performance of hybrid networks combining LoRa and ZigBee, demonstrating the potential for heterogeneous network integration. Bravo-Arrabal et al. [20] implemented hybrid WSNs for disaster response applications, comparing ZigBee and LoRa performance in scenarios involving both stationary and mobile nodes. Leonardi et al. [21] proposed a scheme using LoRa as a method to connect clusters of BLE networks to overcome the short transmission range of BLE and the high latency of LoRa. Sisinni et al. [22] proposed a method to improve the reliability of emergency data transmission while maintaining the existing backend of LoRaWAN by combining LoRaWAN and BLE mesh. This study showed that LoRaWAN enables stable data transmission even in indoor environments with weak signals, and minimizes data loss in emergency situations by supplementing the lack of multi-hop transmission function of LoRaWAN with BLE mesh. Ayele et al. proposed a wildlife monitoring system leveraging both BLE and LoRa to improve energy efficiency and reliability by selecting communication technology based on transmission distances. Similarly, Ferreira et al. [26] combined LoRa and BLE technologies to enhance IoT system performance in smart cities. Additionally, Pérez et al. [24] proposed a fire alarm system utilizing hybrid BLE–LoRa networks to increase transmission efficiency, while Singh et al. [27] employed LoRa and ZigBee in fire alarms and evacuation routes. This system used LoRa for fire alarms and Zigbee for short-range communication to indicate evacuation routes, and measured the performance of each communication technique through simulation.

As mentioned above, research has been actively conducted on communication techniques combining LoRa with BLE or ZigBee to enhance communication efficiency. These studies generally enhance transmission efficiency by simultaneously employing both communication methods. However, most of them do not consider large-capacity data transmission or data characteristics, as they use LoRa to transmit information from clusters composed of Zigbee or BLE over long distances, or use separate transmission technologies when transmitting short-distance data and long-distance data. Moreover, these approaches typically assume environments where periodic data transmission without considering energy harvesting and allocation, which can lead to energy depletion or inefficient use of surplus energy.

To address these limitations, we propose a novel technique that adjusts data collection based on energy harvesting and allocation in environments utilizing various types of data. The proposed method increases both general sensor data and urgent data collection amounts and enhances transmission reliability by sending urgent messages through multiple routes.

## 3. Dual-Mode Data Collection for Periodic and Urgent Data Transmission

### 3.1. Overall System Operation

**Figure 1:**
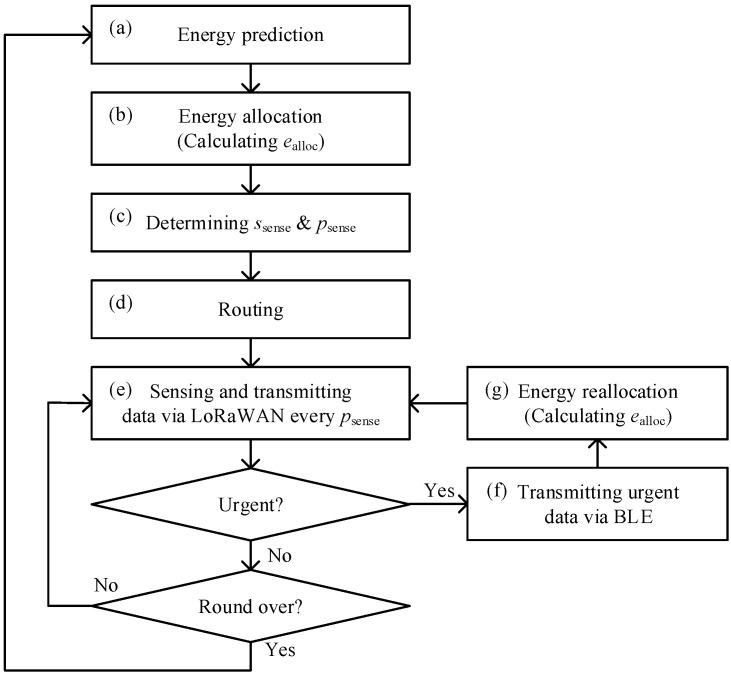
Process diagram of the proposed scheme.

In the proposed scheme, sensor nodes operate on battery power, making their energy resources limited. To overcome this limitation, sensor nodes harvest environmental energy, such as solar power, but due to the restricted amount of energy harvested, efficient energy utilization is essential. The proposed scheme employs an energy harvesting prediction scheme [28] and an energy allocation scheme [14]. The sensor nodes use the energy harvesting prediction scheme to estimate the amount of energy harvested during each operational round. Since the amount of harvested energy, such as solar energy, significantly varies over time, the sensor nodes apply an energy allocation scheme to evenly distribute energy usage over time, thus ensuring uniform data collection. The periodically collected environmental data are transmitted using LoRaWAN, utilizing this allocated energy. In addition, it is necessary to maintain a certain amount of reserved energy at all times for urgent data transmission because sensor nodes should intermittently transmit large amount of urgent data using BLE. To achieve these functionalities, the proposed method follows the process below, with each step illustrated in  Figure 1:

(a) Predict the amount of energy harvested at the beginning of each round using the energy harvesting prediction scheme [28].
(b) Determine the amount of energy $e_{\mathrm{alloc}}$ to be allocated for usage in the current round by employing the energy allocation scheme [14].
(c) Within $e_{\mathrm{alloc}}$, determine the maximum size of sensing data $s_{\mathrm{round}}$ and sensing interval $p_{\mathrm{sense}}$ using the method described in Section 3.3.
(d) Perform routing to determine the path to transmit urgent data via BLE using the modified minimum depth tree (MDT) algorithm described in Section 3.4.
(e) Collect and transmit sensing data every $p_{\mathrm{sense}}$.
(f) On occurrence of urgent data, transmit it through the determined route in (d).
(g) To ensure balanced energy consumption over time, sensor nodes that have transmitted urgent messages reallocate their energy as in (b).

### 3.2. Energy Prediction and Allocation of a Sensor Node

**Figure 2:**
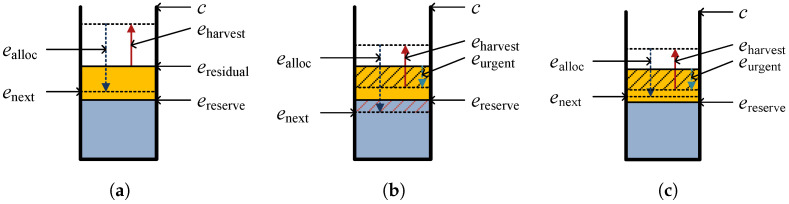
Energy model of a sensor node: (**a**) before sending urgent data, (**b**) after sending urgent data, and (**c**) energy reallocation.

Sensor nodes utilize energy prediction and allocation techniques to evenly distribute energy consumption regardless of time variation. Therefore, we employ ProEnergy [28] as the energy prediction technique and adapt the allocation method proposed by Noh et al. [14]. However, each sensor node reserves a specific amount of energy, denoted as $e_{\mathrm{reserve}}$, for urgent data transmission. Unlike conventional energy allocation techniques, which consider minimum energy $e_{\min}$, battery capacity c, and remaining energy $e_{\mathrm{remain}}$, our approach replaces $e_{\min}$ with $e_{\mathrm{reserve}}$ for energy allocation. Therefore, whereas the conventional methods determine the usable energy and battery capacity as $e_{\mathrm{remain}}-e_{\min}$ and c, respectively, we define them as $e_{\mathrm{remain}}-e_{\mathrm{reserve}}$ and $c-e_{\mathrm{reserve}}$. Consequently, the allocated energy $e_{\mathrm{alloc}}$ for periodic operations within a round can be calculated accordingly.  Figure 2 represents the energy model of the sensor node.

### 3.3. Sensing Period Determination

Sensor nodes periodically transmit sensed data to a sink node. To maximize the data gathered during a round, the maximum sensing and transmission period achievable within the allocated energy $e_{\mathrm{alloc}}$ must be determined. Since sensor nodes can use only $e_{\mathrm{alloc}}$ for each round, the consumed energy must not exceed this limit. According to the energy model proposed by Melodia et al. [29], the consumed energy includes transmission energy $e_{\mathrm{Tx}}$, reception energy $e_{\mathrm{Rx}}$, and other electronic circuit energy $e_{\mathrm{elec}}$. Therefore, the following condition must be satisfied:

(1) $$e_{\mathrm{alloc}}\geq e_{\mathrm{Tx}}+e_{\mathrm{Rx}}+e_{\mathrm{elec}}.$$

Since $e_{\mathrm{Rx}}$ and $e_{\mathrm{elec}}$ are nearly identical for each node, energy consumption should be adjusted by $e_{\mathrm{Tx}}$. Solving for $e_{\mathrm{Tx}}$, we have:

(2) $$e_{\mathrm{Tx}}\leq e_{\mathrm{alloc}}-e_{\mathrm{Rx}}-e_{\mathrm{elec}}.$$

Additionally, $e_{\mathrm{Tx}}$ varies depending on transmission distance r and the amount of data transmitted s, defined as:

(3) $$e_{\mathrm{Tx}}=s\beta r^{\alpha },$$

where β represents the energy consumed per bit per unit distance ($J/\mathrm{bit}/m^{\alpha }$), and α represents the path loss exponent (typically ranging from 2 to 5) [29]. To determine the data amount $s_{\mathrm{round}}$ that can be transmitted in one round, we substitute Equation (3) into Equation (2), yielding:

(4) $$s_{\mathrm{round}}\leq \frac{e_{\mathrm{alloc}}-e_{\mathrm{Rx}}-e_{\mathrm{elec}}}{\beta r^{\alpha }}.$$

Given that each sensor node transmits data sized $s_{\mathrm{unit}}$ per collection, the maximum number of sensing operations per round is $\left\lfloor s_{\mathrm{round}}/s_{\mathrm{unit}}\right\rfloor$, and the sensing period $p_{\mathrm{sense}}$ can be determined as:

(5) $$p_{\mathrm{sense}}=\frac{p_{\mathrm{round}}}{\left\lfloor \frac{s_{\mathrm{round}}}{s_{\mathrm{unit}}}\right\rfloor }.$$

Thus, by collecting and transmitting data at intervals of $p_{\mathrm{sense}}$, sensor nodes can maximize data collection uniformly regardless of time within the allocated energy $e_{\mathrm{alloc}}$.

### 3.4. Routing for Urgent Data

LoRa-based data collection employs single-hop communication, thus not requiring routing. However, BLE communication for urgent data involves short-range transmissions incapable of directly reaching the sink node. Therefore, multi-hop transmission through relay nodes is necessary, requiring route determination. To prevent message loss due to relay node failures, the proposed scheme employs simultaneous transmission via redundant routes. A modified (MDT) algorithm is employed to reduce the energy consumption of relay nodes and improve reliability. Unlike traditional MDT routing [30], which determines a single parent node for forwarding, the proposed method does not enforce strict next-hop selection. Instead, it establishes multiple redundant paths based solely on hop count, allowing sensor nodes to broadcast urgent messages along several routes.

**Figure 3:**
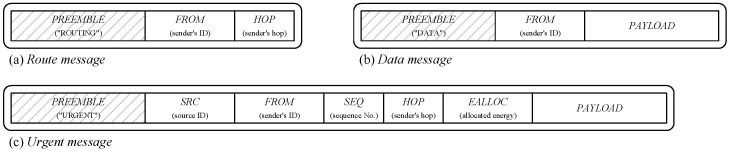
Message formats.

The following describes the modified MDT routing process employed in the proposed scheme. At the start of each round, the sink node broadcasts a *route message* (shown in  Figure 3a), triggering route establishment. Upon receiving the message, nodes update their routes accordingly and propagate the message to neighboring nodes. The detailed modified MDT routing procedure is as follows:

1. The sink node generates and broadcasts a *route message*, setting *HOP* to 0 and *FROM* to its own ID.
2. Nodes receiving the *route message* act based on the following rules:
  (a) If receiving the message for the first time in the current round, the node sets the message’s *HOP* to its h.
  (b) If the node has already determined its h but receives another message, it updates h as the message’s HOP+1 if its value is greater than the message’s HOP+1.
  (c) If the node updates its route, it generates a new *route message* with its h and ID, and broadcasts it.
3. The routing process concludes once no further *route messages* require processing.

### 3.5. Sensor Node Operation for Urgent Data

When transmitting urgent messages, sensor nodes use BLE multi-hop communication. Conventional WSN methods transmit through a single route, risking message loss if any relay node fails. The proposed method enhances redundancy by transmitting urgent messages simultaneously along multiple paths. The detailed node operation is as follows:

1. Node $v_{i}$ with ID *i* broadcasts an urgent message to their neighbor nodes. In the urgent message, *SRC* and *FROM* contain its ID, *SEQ* contains the urgent event number, *HOP* contains its h, and *EALLOC* contains its $e_{\mathrm{alloc}}$.
2. A receiving node $v_{j}$ examines the *SRC* and *SEQ* fields to check if the message is a duplicate. The node then decides whether to relay the message based on the following conditions:
  (a) If the message has already been received, or if the *HOP* is greater than its own $h_{j}$, the node ignores the message.
  (b) If the message is new, but the *HOP* is equal to $h_{j}$, the message is sent by its sibling. In this case, if its $e_{\mathrm{alloc}j}$ is less than or equal to *EALLOC*, it ignores the message because its energy is relatively low to maintain energy balance.
  (c) If the message is new, but the *HOP* is smaller than $h_{j}$, the message is ignored because it is transmitted in a direction away from the sink node.
  (d) Otherwise, the node broadcasts a new urgent message with its own ID, $h_{j}$, and $e_{\mathrm{alloc}}$ in the *FROM*, *HOP*, and *EALLOC* field, respectively.
3. This process repeats until all messages are sent.
4. All nodes that sent the urgent message perform energy allocation to recalculate $e_{\mathrm{alloc}}$.

In this process, nodes compare hop counts to ensure messages are transmitted towards the sink node. Additionally, by comparing $e_{\mathrm{alloc}}$, nodes with lower available energy avoid unnecessary transmissions to maintain energy balance. This mechanism also prevents transmission cycles in routing paths. After transmitting urgent messages, sensor nodes recalculate energy allocation to replenish the energy consumed by urgent message transmission, thereby preventing energy depletion. Algorithm 1 describes this procedure in detail.


**Algorithm 1** Urgent data transmission process**Require:** Node $v_{i}$ senses urgent situation.
   1:$v_{i}$ Broadcasts *urgent message* including *SRC*, *FROM*, *ID*, *SEQ*, *HOP*, and *ELLOC*.   2:**repeat**   3:    Node $v_{j}$ receives *urgent message* from $v_{i}$.   4:    **if** $v_{j}$ already received the *urgent message* with *SRC* and *SEQ*, or $\mathrm{HOP}>h_{j}$ **then**   5:        Ignore the *urgent message*.   6:    **else if** $\mathrm{HOP}=h_{j}$ and $EALLOC\geq e_{\mathrm{alloc}j}$ **then**   7:        Ignore the *urgent message*.   8:    **else if** $\mathrm{HOP}<h_{j}$ **then**   9:        Ignore the *urgent message*. 10:    **else** 11:        $v_{j}$ broadcast a new *urgent message* with its ID, $h_{j}$, and $e_{\mathrm{alloc}j}$. 12:    **end if** 13:**until** All messages have been sent.



**Figure 4:**
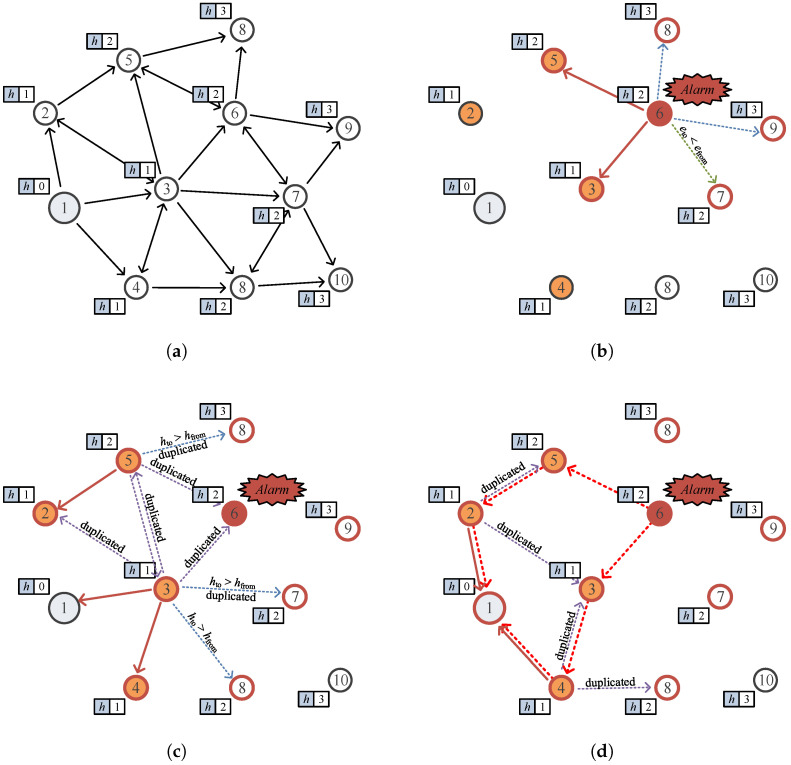
Example of the proposed scheme: (**a**) routing to determine h hop distance to the sink node, (**b**) sending urgent message via BLE, (**c**) node 3 and 5 relay the emergency message, and (**d**) the emergency message arrives at the sink node.

Figure 4 illustrates an example scenario of the proposed scheme. Assume that at the beginning of a round, each node determines its hop count (h) to the sink node through routing, as shown in Figure 4a. In Figure 4b, suppose an urgent event occurs at node 6, which then broadcasts an *urgent message* to inform the sink node. Neighboring nodes (nodes 3, 5, 7, 8, and 9) receive this message and decide whether to relay it or not. Nodes that received the urgent message are marked with red borders in the figure to visually distinguish them from non-receiving nodes. Nodes 8 and 9 ignore the message because their h counts are higher than the sender’s. Node 7 ignores the message due to having less $e_{\mathrm{alloc}}$ than the sender, in order to maintain energy balance. Node 3 relays the message because its h count is lower than that of the sender. Node 5, although having the same hop count as the sender, relays the message because its allocated energy is higher, thus increasing route redundancy. Then, as depicted in Figure 4c, nodes 2, 3, 6, and 8 receive the message transmitted by node 5. Nodes 3, 6, and 8 discard this message as it is duplicated, while node 2 forwards it because its h is smaller than that of node 5. The message transmitted by node 3 is received by nodes 1, 2, 4, 5, 6, 7, and 8. Among these, nodes 2, 5, 6, and 7 ignore the message as it is duplicated, and node 8 discards it due to a higher h compared to the sender. Node 4 forwards the message since its h is smaller than that of the sender, and node 1, being the sink node, successfully receives the *urgent message*. Finally, as shown in Figure 4d, the messages transmitted by nodes 2 and 4 reach the sink node, resulting in a total of three *urgent messages* successfully delivered to the sink node.

Although this approach might consume more energy from multiple sensor nodes due to transmission over redundant paths, urgent events occur infrequently, and each message is transmitted by multiple nodes rather than repeatedly by a single node. Thus, it mitigates energy imbalance among nodes.

## 4. Performance Evaluation

To evaluate the performance of the proposed method, we conducted simulations under various network conditions. The simulations were performed using SolarCastalia [31], an OMNeT++-based simulation framework that extends the original Castalia simulator with support for energy harvesting models, including solar energy prediction. This environment was chosen for its ability to provide fine-grained control over energy consumption parameters, support both BLE and LoRaWAN communication models, and simulate wireless sensor network behavior realistically under energy-constrained scenarios. All simulation logic and configurations were implemented using C++ and NEC (NEtwork Description), the native languages for extending OMNeT++ modules.

We compared the proposed scheme with three alternative approaches: (1) a LoRaWAN-only scheme (*LoRaWAN*), (2) a BLE-only scheme (*BLE*), and (3) the energy-aware method proposed by Ayele et al. [23] (*WMS*). Performance evaluation was based on two metrics: (i) the total number of sensory data packets received at the sink node, and (ii) the arrival rate of urgent data.

The simulated WSN consisted of 200 energy-harvesting sensor nodes and a single sink node, randomly deployed in the field. This node density was selected because it offers a balanced and representative scenario for comparing different schemes. When the number of nodes is significantly lower than 200, energy consumption per node remains low, resulting in small differences among schemes and greater sensitivity to environmental variations. Conversely, increasing the number of nodes beyond 200 leads to excessive data transmission relative to the harvested energy, causing frequent blackouts and making performance comparison unreliable. Therefore, 200 nodes were chosen as a practical configuration to highlight the impact of each scheme under consistent and fair conditions. While the *LoRaWAN*, *BLE*, and *WMS* methods collect and forward sensory data periodically, the proposed method adaptively adjusts the sensing interval according to each node’s energy availability. Urgent messages were generated at random times within predefined intervals. Table 1 summarizes the key simulation parameters used in this study.

### 4.1. Performance Comparison by Number of Nodes

**Figure 5:**
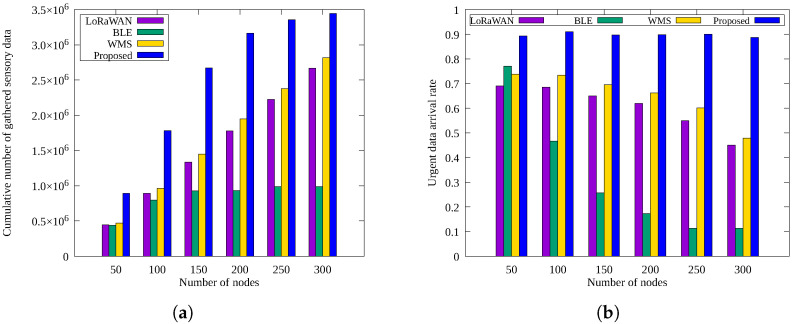
Comparison by the number of nodes: (**a**) cumulative number of sensory data gathered and (**b**) urgent data arrival rate.

Figure 5a,b shows the cumulative number of sensory data packets and urgent data arrival rates at the sink node, respectively, as the total number of sensor nodes increases.

As shown in Figure 5a, the total amount of data collected generally increases with the number of nodes. However, the *BLE* method shows little increase beyond 150 nodes because the increased number of nodes leads to more hops, greater relay load, and higher energy consumption at nodes closer to the sink, resulting in energy depletion. In contrast, *LoRaWAN*, *WMS*, and the proposed scheme successfully scale data collection with increasing node numbers. The proposed scheme collects the most data by adaptively adjusting the sensing rate according to the allocated energy. Although *WMS* performs well by switching between *LoRaWAN* and *BLE*, its fixed data collection rate results in lower performance compared to the proposed scheme with adaptive energy allocation and data collection rate control approach.

In Figure 5b, *LoRaWAN* receives approximately 70% of urgent messages with 50 nodes, but performance decreases as the node count grows due to LoRaWAN’s high Time-on-Air (ToA). *WMS* exhibits similar performance to *LoRaWAN* but slightly better due to adaptive use of BLE. The *BLE* approach initially performs better than *LoRaWAN* at 50 nodes but rapidly deteriorates as node count increases, due to frequent relay node blackouts from energy depletion. The proposed scheme maintains consistent urgent message arrival rates regardless of the number of nodes by reserving energy for urgent transmissions and employing multiple redundant routes.

### 4.2. Performance Comparison by Urgent Message Size

**Figure 6:**
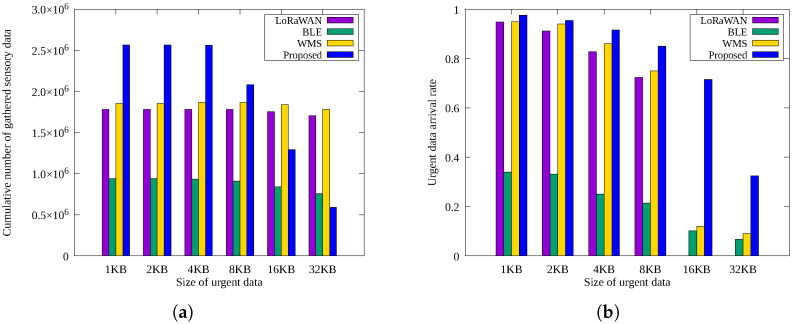
Comparison by the size of urgent data: (**a**) cumulative number of sensory data gathered and (**b**) urgent data arrival rate.

Figure 6a,b presents the cumulative number of sensory data and urgent data arrival rates, respectively, as urgent message size varies.

In Figure 6a, the sensor data collected by *LoRaWAN* and *WMS* remain nearly constant irrespective of urgent message size, because they periodically transmit a constant number of sensory data. However, as shown in Figure 6b, their urgent data delivery rates drastically decrease as message size grows due to higher ToA. Specifically, *LoRaWAN* fails completely at message sizes above 16 KB, while *WMS* shows moderate performance by occasionally utilizing BLE.

The *BLE* method maintains the capability of delivering large urgent messages despite limited sensory data throughput. The proposed scheme achieves the highest urgent data arrival rate through redundant transmission paths. However, due to increased energy expenditure from frequent urgent message transmissions, the proposed scheme reduces periodic sensory data collection as urgent message size increases. Nevertheless, it significantly outperforms other methods in delivering urgent data.

### 4.3. Performance Comparison by Packet Error Rate

**Figure 7:**
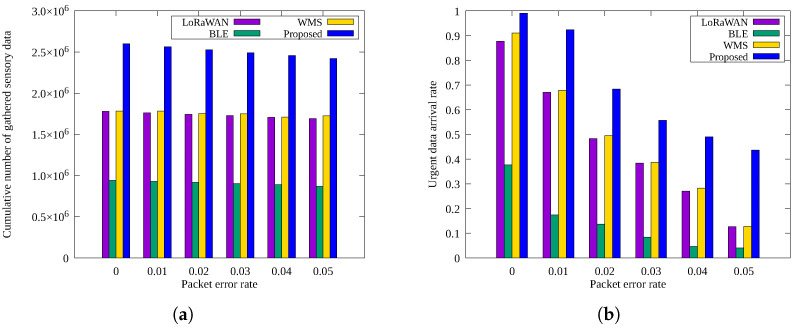
Comparison by the packet error rate: (**a**) cumulative number of sensory data gathered and (**b**) urgent data arrival rate.

Figure 7a,b shows the cumulative number of sensory data and urgent data arrival rates, respectively, according to packet error rate. No error-correction methods (such as ARQ or FEC) were applied in these experiments.

As shown in Figure 7a, packet error rate has minimal impact on the number of sensory data packets collected. However, in Figure 7b, the urgent data arrival rate significantly declines with increasing packet errors. This is because larger urgent data packets are more susceptible to packet loss. Urgent data are large in size, and so must be divided into several packets to be sent; if even one of them is lost, sending fails. Therefore, the arrival rate drops significantly if the error rate increases even slightly. In particular, in the case of *BLE*, which uses multi-hop transmission, the arrival rate is much lower than in other techniques because multiple nodes must relay the data. The proposed scheme maintains a high urgent message delivery rate due to redundant transmissions over multiple paths. If an error recovery technique such as FEC is applied to the proposed scheme, it is expected that the success rate can be further increased and the loss of urgent messages can be minimized.

### 4.4. Performance Comparison by Urgent Message Generation Period

**Figure 8:**
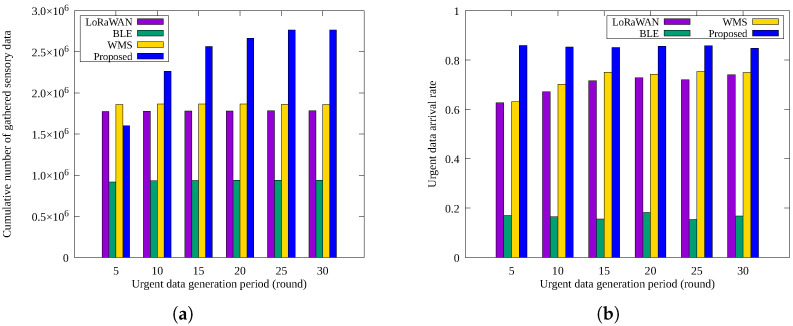
Comparison by urgent data generation period: (**a**) cumulative number of sensory data gathered and (**b**) urgent data arrival rate.

Figure 8a,b shows the cumulative number of sensory data and urgent data arrival rates, respectively, as the frequency of urgent message generation changes. Urgent messages were generated once every 5, 10, 15, …, 30 rounds. Since one round corresponds to approximately one hour in our simulation model, these intervals represent urgency levels ranging from 5 to 30 h. This range was chosen to simulate a variety of real-world scenarios, from frequent minor events (e.g., movement or noise) to rare but critical emergencies (e.g., fire or structural damage). We also observed that when the interval exceeds 30 rounds, the performance differences between schemes become negligible. Therefore, the 5–30 round range was selected as a meaningful and practical test window to assess system responsiveness under varying urgency levels.

As urgent message frequency increases, the proposed scheme collects fewer sensory data packets due to higher energy consumption for redundant urgent message transmissions. For example, at high frequencies (e.g., once within 5 rounds), the proposed method collects less data than *LoRaWAN* or *WMS* because it consumes much energy by repeatedly sending frequent urgent messages through multiple routes. This is a limitation that needs future improvement.

Despite this limitation, as shown in Figure 8b, the proposed scheme consistently maintains a higher urgent message delivery rate. The other methods are largely unaffected by changes in urgent message frequency because these events affect only a subset of nodes. *BLE*’s multi-hop transmission consistently results in lower delivery rates.

## 5. Conclusions

In this paper, we proposed an energy-efficient data collection scheme utilizing both LoRaWAN and BLE in an energy harvesting WSN environment. Sensor nodes periodically collect small-sized environmental data such as temperature, humidity, and light intensity, which are transmitted via LoRaWAN using a single-hop, energy-efficient method. In contrast, large urgent data messages are generated intermittently when abnormal events are detected, such as image capture triggered by intrusion or real-time audio recording, and are transmitted using BLE over multi-hop paths with redundant routes. These urgent transmissions consume reserved energy to ensure fast and reliable delivery. The proposed scheme manages energy by reserving sufficient energy for urgent transmissions, adjusting sensing rates adaptively based on available harvested energy, and using redundant transmission paths to enhance urgent message delivery. Simulation results demonstrate that the proposed scheme significantly increases the amount of collected sensor data and improves urgent message delivery rates compared to existing methods under various conditions. In the future, we plan to explore further improvements, such as integrating error-correction techniques like FEC to enhance urgent message reliability and mitigating the energy consumption challenges caused by redundant urgent transmissions, thereby improving overall sensor data throughput.

## Figures and Tables

**Table 1:** Simulation parameters.

Parameters	Values
Number of nodes	200
Node density	0.04
Duration of a round	1 h
Battery capacity	110 mAh
Sensory data size	20 bytes
Urgent data size	8 kbytes
BLE data rate	250 kbps
LoRaWAN data rate	5.47 kbps
LoRaWAN spreading fector	7
LoRaWAN bandwidth	125 kHz
Transmission power	2 dBm
Sleep power	3.3 µW

## Data Availability

The data presented in this study are available on request from the corresponding authors.

## Abbreviations

The following abbreviations are used in this manuscript:

BLE	Bluetooth Low Energy
LoRa	Long Range
LoRaWAN	Long Range Wide-Area Network
MDT	Minimum depth tree
ToA	Time on Air
WSN	Wireless sensor network
WPT	Wireless power transfer
RF	Radio frequency
